# Obstetric Outcomes of Eritrean Immigrants in Switzerland: A Comparative Study

**DOI:** 10.3389/ijph.2024.1606745

**Published:** 2024-05-08

**Authors:** Rahel M. Erhardt, Kristen Jafflin, Nejimu Zepro, Charles Abongomera, Afona Chernet, Daniel Henry Paris, Sonja Merten

**Affiliations:** ^1^ Swiss Tropical and Public Health Institute, Basel, Switzerland; ^2^ University of Basel, Basel, Switzerland

**Keywords:** Eritrean immigrants, obstetric outcomes, Switzerland, emergency C-section, analgesia

## Abstract

**Objectives:** This study aims to compare obstetric outcomes between Eritrean and Swiss women in Switzerland, focusing on instrumental or surgical interventions and analgesia use.

**Methods:** The study included data from 45,412 Swiss and 1,132 Eritrean women who gave birth in Swiss hospitals (2019–2022). Mixed-effects logistic regression was used to assess the effect of nationality on mode of delivery and analgesia use and multinomial mixed-effects logistic regression to assess the effect of nationality on mode of delivery in women intended for spontaneous vaginal delivery.

**Results:** Compared with Swiss, Eritrean women had a lower rate of primary C-section (Adj. OR 0.73, 95% CI [0.60, 0.89]) but a higher risk of initially planned vaginal deliveries ending in emergency C-section (RRR 1.31, 95% CI [1.05, 1.63]). Eritrean women were less likely to receive epidural analgesia (Adj. OR 0.53, 95% CI [0.45, 0.62]) and more likely to not receive any analgesia (Adj. OR 1.73, 95% CI [1.52, 1.96]).

**Conclusion:** This study reveals disparities in obstetric care, notably in higher emergency C-section rates and lower analgesia use among Eritrean women. For promoting equitable healthcare practices deeper understanding of obstetrics decision-making is needed.

## Introduction

In recent years, Eritrean asylum seekers have become one of the largest groups in Switzerland, with over 40,000 Eritrean immigrants currently residing in the country [1]. It’s important to recognize that women immigrating from sub-Saharan African (SSA) often have distinctive sexual reproductive health (SRH) needs compared to the host population [2–4]. Understanding and addressing these specific SRH needs among the immigrant populations in Switzerland becomes increasingly vital, as they constitute an integral part of society.

Previous research examining obstetric complications among women from SSA countries seeking asylum in the Global North has presented a complex landscape of findings. Some studies indicated an elevated risk of obstetric complications such as low birth weight (LBW) [2, 5], increased rates of meconium and episiotomies, as well as preterm deliveries [6], along with a heightened risk for perinatal and neonatal mortality and stillbirths [7]. Conversely, other findings have indicated a reduced likelihood of preterm delivery [8, 9] and LBW [2, 8, 9]. The concrete reasons for these divergent observations remain unclear.

However, the root causes of these health disparities among immigrant women, when observed, are the result of a complex interplay of factors. Immigrant women often face multiple risks in their home countries and during their journeys that can have detrimental effects on their pregnancy outcomes. These risks include a heightened vulnerability to communicable diseases, including sexually transmitted infections, a reliance on unsafe abortion practices, and a lack of access to healthcare before and after childbirth [3].

Even after their arrival in a new country, immigrant women grapple with stressors such as low socioeconomic status, language barriers, and limited transcultural healthcare knowledge among healthcare providers [4, 10, 11]. These factors have been shown to serve as barriers to accessing healthcare services and ultimately can contribute to poorer obstetric outcomes. Yet, our understanding of obstetric complications among Eritrean women in Switzerland remains very limited.

Another factor that has been shown to affect women’s pregnancy outcomes is female genital cutting (FGC). FGC is a term referring to different severity of surgical removal of the external female genitalia and is categorised into four main types, with type 3, also known as infibulation, being the most invasive one [12]. Eritrea, alongside Somalia and Sudan, has one of the world’s highest FGC rates, estimated at around 82% [12]. While there is no precise data available on the prevalence of FGC among Eritrean women in Switzerland, indirect estimates for women and girls aged over 15 suggested that there were over 13,000 cases in Switzerland in 2018 [13].

In several countries in the Global North, women who have undergone FGC have been shown to be at greater risk of having a C-section [8, 14–17]. In Switzerland, research has shown differences in C-section rates in hospitals based on the nationality of the mother, with Somali women having similar or higher rates than Swiss women [18]. However, other findings suggest that women from African countries with high FGC prevalence may have equal or lower C-section delivery rates compared to women from the Global North [8]. The exact reasons for these variations remain unclear. It is possible that social and cultural norms in the country of origin and destination about childbirth play a role in these findings [19]. The different types of FGC among women could be another contributing factor, as research indicates that obstetric complications differ across FGC types, with infibulation presenting the highest risk of birth complications and C-section [20, 21]. Yet, despite the growing number of Eritrean women in Switzerland, information regarding C-section rates is currently lacking.

In addition to an increased risk of obstetric complications, women who have undergone FGC often report experiencing extreme pain during labour [22, 23]. However, the existing literature paints a mixed picture of current obstetric practices among various immigrant groups, including those with high estimated prevalence of FGC. For instance, a study in Norway revealed significant differences in the use of epidural analgesia among immigrants, with lower rates observed among Somali women [24]. In contrast, a recent study conducted in France did not find such differences [25].

One hypothesis that has surfaced from qualitative research with women from SSA, including Eritrea, is that these women may reject pain relief during childbirth based on their belief that labour pain is an essential and innate part of the childbirth experience [26, 27]. This belief is accompanied by various fears expressed by women from this region about analgesia, such as: i) increasing the risk of C-sections [28], ii) disrupting the natural birth process and slowing down delivery, as pain is associated with immediate delivery [17], iii) making the baby sleepy or harming the baby [26].

However, it’s important to note that pain management during childbirth is not solely determined by the birthing women but also by the healthcare staff. In the United States, racial prejudice has been suggested to influence the use of epidurals during labour [29]. Furthermore, it is well-established that the administration of analgesia necessitates clear explanations and effective communication [6]. It’s plausible that racial stereotypes of healthcare staff towards women from SSA and language barriers might contribute to differences in obstetric practices.

Research over the past two decades has shown that women with FGC who have migrated to the Global North rarely have a voice in procedures related to childbirth and pain management, despite their advocacy for informed choice, respectful treatment, and the competence of medical staff in treating women with FGC [30, 31]. In Switzerland, qualitative research yielded similar findings suggesting that migrant women from countries with high FGC prevalence have limited involvement in decision making regarding obstetric interventions during labour and delivery and report instances of disrespectful care [32, 33]. This carries a danger of re-traumatization during childbirth especially among vulnerable women [30].

In Switzerland, there is limited information about the mode of delivery and pain management during labour in women who have fled countries with a high prevalence of FGC. This study aims to develop a better understanding of obstetric outcomes of Eritrean women in Switzerland, particularly with regard to instrumental/surgical interventions and pain management during labour, and to compare outcomes with those of Swiss women. Based on secondary data analysis, we aim to clarify the role of country of origin and language barriers on obstetric practices and contribute to a better understanding of obstetric outcomes in women from countries with a high prevalence of FGC.

## Methods

The findings are based on a secondary data analysis. Initial data were obtained from the Baby Friendly Hospital Initiative (BFHI) monitoring database. The BFHI, established by the World Health Organization (WHO) in 1991, aims to enhance breastfeeding rates through a program implemented in healthcare facilities, resulting in their certification as Baby-Friendly Hospitals. Since 2000, routine data on every birth occurring in Baby-Friendly Hospitals in Switzerland has been systematically collected. A total of 24 hospitals from the German, French and Italian speaking regions of Switzerland, are still included in BFHI. For this analysis, data from 2019–2022 were included. Data comes from all participating hospitals (20–24 depending on the year).

### Maternal, Infant, and Delivery Characteristics

Maternal Health indicators included variables such as anemia, gestational diabetes, HIV status, maternal smoking status, and mental health condition. Child health indicators comprised perinatal death, respiratory distress, hemolysis, hyperthermia, hypoglycemia, birth weight, gestational age of the child, and whether a transfer to the Intensive Care Unit (ICU) occurred. Type of delivery was categorized into four categories spontaneous vaginal, vaginal instrumental, primary C-section and emergency C-section. Analgesia during labour was categorized into no analgesia, epidural analgesia, spinal analgesia, patient-controlled analgesia, general anaesthesia, or other.

Additional data included the mother’s age, her insurance status (categorized as “basic insurance” or “private insurance”), parity (categorized as “first child,” “second child,” and “third or more children”), and multiparous births treated as a binary variable (yes/no). The binary variable oral language comprehension (yes/no), indicating whether oral comprehension is possible, was used to code for language barriers.

### Statistical Analysis

The analysis was completed using the software Stata (16.1). Initially, a descriptive analysis was conducted to compare fundamental maternal characteristics between Swiss and Eritrean women. These characteristics included age, parity, occurrence of multiple births, and insurance status. Differences were assessed using the Chi-squared test, with a significance level set at 0.05.

In the second step, mixed-effect logistic regression analyses were carried out to investigate the associations between Eritrean nationality and various maternal and child health indicators, type of delivery, and the use of analgesia during labour. These regression models were adjusted for potential confounders, including maternal age, parity, occurrence of multiple births, insurance status and random effects for place of delivery. The results are presented as adjusted odds ratios (Adj. OR) with corresponding 95% confidence intervals, with a significance level set at 0.05.

In a third step, multinomial mixed-effect logistic regression analyses were conducted specifically to assess the influence of nationality on the type of delivery for women who initially intended to have a vaginal delivery (excluding women with primary C-section) (Model 1). Two additional models were developed, incorporating language barriers (Model 2) and the presence of gestational diabetes (Model 3). These models were likewise adjusted for maternal age, parity, occurrence of multiple births, insurance status, and random effects for place of delivery. The results are presented as relative risk ratios (RRR) along with their corresponding 95% confidence intervals. Sensitivity analyses were carried out, including additional maternal and neonatal health indicators, such as mental health conditions, high/low birth weight, and gestational age, to assess their potential impact on the models.

## Results

A total of 45,412 Swiss and 1,132 Eritrean women had given birth in the 24 hospitals included in this analysis in the French, Italian and German-speaking parts of Switzerland. Eritrean women were generally younger than Swiss women (30.1 vs. 32.7 years), and less likely having their first child compared to their Swiss counterparts (27.4% vs. 50.9%). All women had either basic insurance or private/semi-private insurance, as per Swiss law. Only two Eritrean women had private or semi-private insurance (0.02%), compared to 9.4% of Swiss women (Table 1).

**TABLE 1:** Maternal characteristics (Data from 24 hospitals in Switzerland, 2019-2022).

	Swiss (N = 45,412)	Eritrean (N = 1,132)	*p*-value
Mean age in years (SD)	32.7 (SD = 0.22)	30.1 (SD = 0.16)	<0.001^#^
1st child	23,064 (50.9)	306 (27.4)	
2nd child	16,338 (36.0)	364 (32.3)	
3rd child	4,606 (10.2)	262 (23.3)	
4th and more children	1,364 (2.9)	195 (17)	<0.001^¬^
Multiple birth	1,196 (2.6)	17 (1.5)	0.018 ^¬^
Language Barriers (No)	45,067 (99.3)	598 (52.8)	
Language Barriers (Yes)	323 (0.7)	534 (47.2)	<0.001^¬^
Total	45,390 (100)	1,132 (100)	
Private insurance	4,289 (9.4)	2 (0.2)	
Basic insurance	41,078 (90.6)	1,129 (99.8)	<0.001^¬^
Total	45,367 (100)	1,131 (100)	

# *p*-value from *t*-test. ¬ *p*-value, Chi2 test.

Information on maternal body mass index (BMI) was not available, but Eritrean women were more likely to have a record of gestational diabetes during the current pregnancy (17.5% compared to 9.0% among Swiss women), did not smoke (except two women), and were rarely diagnosed with a mental health condition during pregnancy or while on the maternity ward (1.2% vs. 2.3% for Swiss women). Eritrean women were also more likely to be HIV positive than Swiss women (0.8% compared to 0.0%) (Table 2).

**TABLE 2:** Maternal and neonatal health conditions: Frequency and association with nationality (Data from 24 hospitals in Switzerland, 2019-2022).

Nationality	Swiss	Eritrean		Eritrean (reference: Swiss)		
Maternal conditions	N (%)	N (%)	*p*-value	Adj. OR^a^	95% CI	*p*-value
Gestational diabetes	4,071 (9.00)	198 (17.5)	<0.001^¬^	2.04	(1.74, 2.41)	<0.001
Smoking	1,522 (3.4)	2 (0.2)	<0.001^¬^	0.03	(0.01, 0.12)	<0.001
Mental health condition	1,332 (2.3)	14 (1.2)	0.001	0.39	(0.23, 0.67)	0.001
Maternal anemia	1,109 (2.5)	19 (1.7)	0.095^¬^	0.81	(0.51, 1.30)	0.385
Maternal HIV	16 (0.0)	9 (0.8)	<0.001^¬^	17.52	(6.96, 44.11)	<0.001

Neonatal health	N (%)	N (%)	*p*-value	Adj. OR^a^	95% CI	*p*-value
Respiratory distress	1,350 (3.0)	39 (3.5)	0.371	1.48	(1.06, 2.06)	0.021
Hemolysis	31 (0.1)	2 (0.2)	0.178	1.49	(0.43, 6.49)	0.597
Hypothermia	378 (0.8)	21 (1.9)	<0.001	2.24	(1.41, 3.56)	0.007
Hypoglycemia	1,687 (3.7)	74 (6.5)	<0.001	1.07^b^	(0.54, 2.12)	0.141
Low birth weight	3,258 (7.2)	56 (5.0)	0.004	0.54^c^	(0.41, 0.73)	<0.001
Very low birth weight	1,632 (3.6)	21 (1.9)	0.002	0.34^c^	(0.22, 0.54)	<0.001
Macrosomia (>4500 g)	279 (0.6)	6 (0.5)	0.721	0.69	(0.30, 1.57)	0.376
Preterm (<37 weeks)	3,440 (7.6)	48 (4.2)	<0.001	0.40	(0.29, 0.54)	<0.001
Very preterm (<32 weeks)	450 (1.0)	10 (0.9)	0.718	0.49	(0.26, 0.94)	0.031
Postterm (>42 weeks)	175 (0.4)	3 (0.3)	0.517	0.87	(0.27,2.76)	0.810
Transfer to ICU	3,111 (6.9)	59 (5.2)	0.060	1.16	(1.00, 1.35)	0.050
Total number of births	45,412 (100)	1,132 (100)				

^a^ Odds ratio from mixed-effect logistic regression analysis controlled for age, parity, multiple birth, insurance status, including random effects for place of delivery (hospital).

^b^ Odds ratio from mixed-effect logistic regression analysis controlled for age, parity, multiple birth, insurance status, including random effects for place of delivery (hospital) using The Berndt-Hall-Hall-Hausman (BHHH) algorithm as optimization technique for maximizing the likelihood function.

^c^ Odds ratio from mixed-effect logistic regression analysis controlled for age, parity, multiple birth, insurance status, maternal diabetes including random effects for place of delivery (hospital).

The children of Eritrean women were less likely to be delivered preterm, have a low birth weight, or to be transferred to the ICU; only hypothermia and hypoglycemia were more common among the neonates of Eritrean mothers, whereby the differences in hypoglycemia was no longer significant after controlling for age, parity, multiple birth, insurance status, including random effects for place of delivery (Table 2).

Table 3 shows differences in mode of delivery and analgesia during labour. Primary C-Sections were significantly less common among Eritrean women (12.7% compared to 16.2% among Swiss women). In addition, Eritrean women show higher risk for emergency C-section, yet values failed to be statistically significant (adjusted odds 1.19, 95% CI 1.00, 1.42). Differences in instrumental deliveries were not found to be statistically different. In addition, the proportion of Eritrean women with spontaneous vaginal births was higher than for Swiss women (65.4% compared to 57.7%), however after controlling for age, parity, multiple births, the odds of having a spontaneous birth were not significantly higher (adjusted odds ratio 1.03, 95% CI 0.90, 1.18).

**TABLE 3:** Mode of delivery and analgesia: Frequency and association with nationality (Data from 24 hospitals in Switzerland, 2019-2022).

	Swiss	Eritrean	*p*-value	Eritrean (reference: Swiss)		
Mode of delivery	N (%)	N (%)	*p*-value	Adj. OR^a^	95% CI	*p*-value
Vaginal, spontaneous	26,201 (57.7)	739 (65.4)	<0.001	1.03	(0.90, 1.18)	0.664
Vaginal, instrumental	4,434 (9.8)	84 (7.3)	0.009	1.17	(0.92, 1.49)	0.193
Emergency C-section	7,439 (16.4)	163 (14.4)	0.078	1.19	(1.00, 1.42)	0.05
Primary C-Section	7,316 (16.2)	144 (12.7)	0.002	0.73	(0.60 0.89)	<0.001

Analgesia	N (%)	N (%)	*p*-value	Adj. OR^a^	95% CI	*p*-value
No analgesia	16,793 (37.0)	612 (54.1)	<0.001	1.73	(1.52, 1.96)	<0.001
Patient controlled snalgesia	1,158 (2.6)	25 (2.1)	0.471	0.87	(0.56, 1.35)	0.538
Epidural anesthesia	13,945 (30.7)	191 (16.9)	<0.001	0.53	(0.45, 0.62)	<0.001
Spinal anesthesia	10,989 (24.2)	232 (20.5)	0.004	0.86	(0.73, 1.00)	0.047
General anesthesia	1,038 (2.3)	28 (2.5)	0.667	1.01	(0.68, 1.50)	0.943
Other (inhaled analgesia	1,414 (3.1)	41 (3.6)	0.332	1.23	(0.88, 1.70)	0.219
synthetic opioid)						
no answer	57 (0.1)	3 (0.3)	0.196			
Total number of women	45,412 (100)	1,132 (100)				

^a^ Odds rati from mixed-effect logistic regression analysis controlled for age, parity, multiple birth, insurance status; including random effects for place of delivery (hospital).

No difference was found for general anesthesia. Eritrean women were significantly less likely receiving epidural analgesia during labour than Swiss women (16.9% compared to 30.7%), and spinal analgesia for C-section was also less common among Eritrean women (20.5% overall compared to 24.2%, respectively). Overall, Eritrean women were significantly less likely to receive any analgesia at all (adjusted odds ratio 1.73, 95% CI 1.52, 1.96). We examined the influence of language barriers on analgesia use and found no statistically significant relationships. Results of these analyses can be found in our Supplementary Material (Supplementary Table S1).

We were further interested in the extent to which language barriers or medical conditions (i.e., gestational diabetes) could explain the difference in rate of instrumental vaginal births or emergency C-sections, outcomes that suggest a need to resort to additional measures in a birth that was initially planned to be a spontaneous vaginal birth (Table 4). Eritrean women had a higher risk of experiencing an instrumental delivery (vacuum) (RRR 1.31, 95% CI 1.05, 1.63) or emergency C-section (RRR 1.33, 95% CI 1.13, 1.57) when adjusting for maternal age, parity, multiple births, insurance status and effects for place of delivery (Model 1). However, the associations were no longer significant when a dichotomous variable indicating language barriers was added to the model (Model 2). This remained unchanged after the addition of a variable for gestational diabetes (Model 3). No control variables were associated with an increased risk of instrumental delivery in models two and 3, whereas language barriers (model 2) and language barriers and gestational diabetes (model 3) were each associated with higher risks of emergency C-section.

**TABLE 4:** Outcomes of instrumental or emergency C-section in Eritrean versus Swiss women planning spontaneous vaginal delivery (excluding primary C-section) (Data from 24 hospitals in Switzerland, 2019-2022).

	Model 1		Model 2		Model 3	
	RRR^a^	95% CI	RRR^a^	95% CI	RRR^a^	95% CI
Spontaneous vaginal delivery (Reference)	1		1			
Instrumental delivery (Ref: Spontaneous)						
Eritrean nationality	1.31	(1.05, 1.63)*	1.19	(0.85, 1.66)	1.19	(0.85, 1.67)
Language barriers		-	1.23	(0.83, 1.81)	1.23	(0.84, 1.81)
Gestational diabetes		-		-	1.01	(0.89, 1.14)
Emergency C-Section (Ref: Spontaneous)						
Eritrean nationality	1.33	(1.13, 1.57)**	1.13	(0.91, 1.42)	1.10	(0.88, 1.35)
Language barriers		-	1.40	(0.91, 1.42)**	1.39	(1.14, 1.70)**
Gestational diabetes		-			1.44	(1.30, 1.59)***

^a^ Relative Rsk Ratio for mixed-effect multinominal logistic regression controlled for age, parity, multiple birth, insurance status; including random effects for place of delivery (hospital). ***p-value<0.001; ** *p*-value<0.01; * *p*-value<0.05; (*) *p*-value<0.1.

## Discussion

The main aim of this study was to investigate differences in obstetric outcomes during childbirth for Eritrean as compared to Swiss women, with a specific focus on the use of instrumental or surgical interventions and the administration of analgesia during labor. Additionally, the research aimed to explore the impact of language barriers on those outcomes.

We found a higher probability of initially planned natural vaginal births among Eritrean women culminating in emergency C-sections, primarily attributed to language barriers and maternal diabetes. Additionally, we noted a pattern of limited utilization of analgesia, including epidural analgesia, among Eritrean women. Overall, Eritrean women were younger than their Swiss counterparts, have given birth to more children, in line with previous research [5]. Conversely to some prior investigations [6, 34], the Eritrean population exhibited fewer instances of preterm births, low and very low birth weights, and neonatal transfers to the ICU when compared to Swiss nationals. However, Eritrean neonates did display elevated rates of hypothermia and hypoglycaemia, while differences in prevalence of hypoglycaemia failed to be statistically significant.

Previous research has shown a significant association between hypothermia and hypoglycaemia, [35]. Notably, this research indicated that nearly 50% of neonates experiencing hypoglycaemia also presented with hypothermia (body temperature <36.5°C). Furthermore, prior work has established an augmented risk of hypoglycaemia among mothers diagnosed with maternal gestational diabetes [36].

The higher rates of hypoglycaemia and hypothermia among Eritrean neonates, along with the elevated prevalence of maternal gestational diabetes in this group, could indicate a potential lack of adequate treatment for maternal gestational diabetes during pregnancy. In other words the negligence in the management of maternal diabetes during pregnancy may be a contributing factor to the increased incidence of hypoglycaemia and hypothermia in new-borns. Yet, further research is needed to support this hypothesis.

This study found that Eritrean women had a lower rate of primary C-sections and a higher likelihood of emergency C-sections. Although, the results for the latter failed to reach statistical significance. There was, however, an increased risk for Eritrean women compared with Swiss women of intended spontaneous vaginal births ending in emergency C-section. Interestingly, this increased risk was primarily attributed to language barriers and gestational diabetes rather than Eritrean nationality. Overall, these findings are in line with prior research in the Netherlands and Israel, which also find high rates of emergency C-sections among Eritrean women as compared to the native population [5, 6]. Similarly, prior studies in Switzerland found higher C-section rates among Somali immigrants than Swiss nationals [18]. Yet, none of these studies examined language barriers or maternal diabetes as potential confounders.

While we acknowldge that language barriers do not equate to communication barriers due to potential translation services, recent quantitative studies conducted in Switzerland in 2019 and 2022 revealed significant deficiencies in professional interpretation services within Swiss primary care settings [37, 38]. According to Buser et al. (2022), of participants with limited language skills almost 90% lacked access to professional interpreters, and two-third of them did not utilize *ad hoc* interpretator such as family members. Additionally, more than half of participants with limited language skills reported experiencing communication barriers. In a qualitative study focusing on maternity care in Switzerland, which involved allophone female immigrants, healthcare professionals, and intercultural interpreters, results showed that in the absence of proper interpretation services, communication was inadequate for adequately informing women about treatment options and addressing their expectations and needs [10].

In the context of these findings, it’s plausible that in the case of women with limited langauge skills and inadequate means of communication, coupled by risk factors such as maternal diabetes (interpreted as an indicator of high BMI), health professionals propt for emergency C-sections over vaginal delivery options in the event of complications. This is particularly important in light of qualitative findings from the United States, Canada, and the United Kingdom, where a strong preference for natural delivery and persistent fears of C-sections have been reported within the Somali community [31, 39–41].

In a study of Somali women in the United Kingdom, Somali women narrated that, in response to their having undergone FGC, health professionals put pressure on these women to opt for a C-section, as they were perceived as unable to deliver naturally, leading to a sense of powerlessness in defending their preference for natural birth [42]. It is essential to highlight that vaginal birth in case of FCG needs well trained healthcare staff along with effective communication [43]. A qualitative study conducted in Switzerland, which examined obstetric practices concerning Eritrean and Somali women who have undergone FGC, observed that C-sections are frequently seen as a standard procedure for these women. This is especially the case in hospitals with limited experience in managing cases of FGC. Furthermore, the research highlighted challenges in communication during obstetric decision-making between healthcare staff and women. And the same study also revealed that Eritrean women expressed apprehension regarding the high prevalence of C-sections [32]. However, if the preferences of women who have undergone FGC were simply disregarded, and if C-sections were routinely administered to these women in Swiss Hospitals, one might have anticipated a higher incidence of primary C-sections among Eritrean women than what was actually observed.

When discussion the potential role of FGC in the observed higher rates of emergency C-sections in this study, is important to acknowledge that depending on the type of FGC, findings tend to differ. In general, there is a scarcity of data distinguishing obstetric complications according to the various types of FGC. In results from Ethiopia only infibulated women had showed significant higher risk of C-section delivery compared to Women who have not undergone FGC [21]. In a study conducted in the United Kingdom comparing obstetric outcomes between women who have undergone FGC and women who have not, over 85% had type I, 15% type II, the study reported none with type III [20]. Findings of that study revealed no elevated risk of C-sections among women undergone FGC.

Our comprehension of the prevalence of various forms of FGC among Eritrean immigrants in the Global North is significantly limited. There is a possibility that Eritrean women primarily undergo type I and type II FGC, which could lead to an absence of increased risk of C-sections resulting from FGC. On the other hand, we would like to propose another hypothesis to explain the observed emergency C-section rates: healthcare providers in Switzerland do consider the natural childbirth preferences of Eritrean women during the planning stage. However, in instances involving FGC and compounded by additional complications such as high BMI, if communication, and potentially training, is insufficient to address these challenges, healthcare staff may opt for emergency C-sections over vaginal delivery options. However, given the lack of data, this hypothesis remains speculative.

To explore this hypothesis future research that looks more deeply at the dynamics of decision making in complex birth processes among immigrant women, including those involving FGC, will be crucial. Nevertheless, the findings in this study raise important questions regarding the extent to which decision-making in complex delivery processes considers the voices of these women, and if Swiss healthcare staff are adequately trained for birth deliveries if cultural and language gaps exist.

Eritrean women in Switzerland were notably less likely to use any form of analgesia during childbirth than Swiss women. Compared with Swiss women, Eritrean women were less likely to choose epidural analgesia during labour, and this difference was not significantly affected by language barriers. In addition, compared with Swiss women, Eritrean women were more likely not to use any form of analgesia during labour, again with no effect of the language barrier on this difference.

Overall, these findings align with previous research indicating that Eritrean women in the Global North use epidural analgesia less frequently and no analgesia more frequently than native women [5, 6, 16]. Various explanations have been proposed to account for the observed differences in the use of analgesia during labour among different nationalities. As mentioned above, qualitative findings with women from SSA have described labour pain as a natural and integral part of childbirth [26, 27]. This perspective has the potential to foster an overly simplistic notion that Eritrean women, as a collective, are less likely to seek pain relief because of their positive outlook on labour pain.

However, a study conducted at a referral hospital in Eritrea, which examined satisfaction with obstetric procedures, revealed a high level of dissatisfaction with pain relief methods [44]. Women reported that childbirth was more painful than anticipated and that they did not receive adequate pain relief methods. Notably, the authors did not further explore the specific type of pain relief methods that women desired or how they perceived pharmaceutical pain relief options like epidural analgesia [44].

Another study that examined analgesia outcomes in Eritrea highlighted a lack of analgesia, a shortage of pain management specialists, and restricted roles for anaesthesiologists, mainly confined to the operating room [45]. Similar findings have been observed in Tigray, Ethiopia, where over 75% of healthcare workers in obstetric units did not consider the use of analgesia necessary for managing labour pain [46]. In another Ethiopian study, the utilization of pharmacological methods by nurses for labour analgesia during childbirth was nearly non-existent [47]. These findings suggest that the administration of pharmaceutical analgesia in maternity wards may be an unfamiliar practice in obstetric care in Eritrea, and consequently, for Eritrean women.

Consistent with the findings of other studies in which fears of pharmaceutical pain relief have been expressed [17, 26, 28], Eritrean women in Switzerland in a qualitative study, expressed particular concerns about the potential side effects associated with epidural analgesia [48]. These concerns included worries about interference with the natural birth process, such as the inability to push or the fear of being unable to walk after receiving epidural analgesia. Interestingly, in that study, most women cited other women within their own communities as their primary source of information about epidural analgesia.

It is crucial to acknowledge that the administration of pain medication during childbirth is not solely determined by the wishes women bring with them into the labour ward but also by those of healthcare professionals [28]. This holds true in Switzerland, where midwives play a significant role in pain management, a role that becomes particularly critical in situations involving language or cultural barriers that may impede understanding.

For instance, in a study by Chalmers in Canada, 30% of Somali women reported having little say in procedures in pain management [31]. In the United Kingdom, Somali women reported their inability to make decisions about pain management due to communication barriers, leaving such decisions in the hands of healthcare providers [43].

It is known that pain perception, response, and expression vary greatly among individuals and depend on cultural background. There is a risk of misinterpreting the situation when healthcare workers make decisions without effectively engaging with the woman, owing to cultural gaps between the woman’s expression of pain and the healthcare worker’s interpretation of it [49]. Findings on pain perception of midwives indicated their assessment of the necessity for pain support during childbirth was influenced by the woman’s nationality [50]. In a prospective study in Israel, medical staff interpreted that women of Bedouin ethnicity experienced less pain compared to patients of Jewish ethnicity, despite recording similar self-reported pain scores [51].

Expanding upon these findings, the outcomes of this study raise concerns regarding whether healthcare professionals in Swiss labor wards have the necessary time, linguistic capabilities, and cultural competences to effectively understand a woman’s perception of labor pain, inform on misconceptions about analgesia, and to offer appropriate pain management that aligns to patients informed wishes.

### Limitations

The prevalence of FGC could only be estimated based on previous findings in the literature as, no information on FGC was collected. Limitations in the data prevented examination of many potential factors of influence. The data included information about parity, but no information was available on previous C-section, spontaneous or induced labour, or fetal presentation. In addition, there were no causes reported for the conduction of emergency C-section. Furthermore, it included an indicator of communication difficulties (language barriers), but no information about whether a professional interpreter was present to discuss the birth with the woman on entry or before. The data included information on nationality, but not length of stay in Switzerland or legal status. As there was no information on education, employment, or income, we were unable to control for socio-economic status.

### Conclusion

In conclusion, this study sheds light on critical aspects of obstetric care between Eritrean and Swiss women, highlighting disparities in the rates of emergency C-section and the use of analgesia during labour. Findings revealed an increased likelihood of initially planned natural vaginal births ending in emergency C-sections of Eritrean women, which was primarily explain by language barriers and maternal diabetes. Given the presumption that Eritrean women prefer spontaneous vaginal delivery, these findings point to the challenges in achieving shared decision-making when these complicating factors for spontaneous vaginal delivery are present. And may lead to the increased rate of emergency C-sections among Eritrean women, potentially exceeding what is truly needed.

We also observed a trend of low uptake of analgesia, including epidural analgesia, among Eritrean women. This is especially worrying in consideration of the existing literature that highlights misconceptions about pharmaceutical pain relief within the Eritrean community and cultural stereotyping among healthcare professionals, which has been shown to influence pain administration. To prevent potential undertreatment of Eritrean women, further qualitative work is needed to determine what conceptions around birth persist within the Eritrean community, and how linguistic and cultural barriers faced by healthcare workers affect obstetric care. In order to advance equitable and patient-centered obstetric practices, the ultimate objective is to use these findings to direct discussions and efforts focused at increasing cultural sensitivity and awareness.
