# Supplementary material for: Obstetric Outcomes of Eritrean Immigrants in Switzerland: A Comparative Study
Source: Int J Public Health. 2024 May 8;69:1606745. doi: 10.3389/ijph.2024.1606745 (PMC11110796; doi:10.3389/ijph.2024.1606745)
Supplement: Supplementary file 1 [file Table1.DOCX]

**Table S1**
Influence of language barriers on ‘No analgesia’ and ‘Epidural Analgesia’

|  | Model 1 |  | Model 2 |  |
| --- | --- | --- | --- | --- |
|  | OR^#^ | 95% CI | OR^#^ | 95% CI |
| **No analgesia** | | | | |
| Eritrean nationality | 1.52 | (1.25, 1.84)*** | 1.46 | (1.21, 1.76)*** |
| Language barriers |  | - | 1.09 | (0.89, 1.3) |
| **Epidural analgesia** | | | | |
| Eritrean nationality | 0.56 | (0.41, 0.77)*** | 0.63 | (0.47, 0.84)* |
| Language barriers |  | - | 0.79 | (0.61, 1.02)(*) |

^#^ Odds ratio from mixed-effect logistic regression analysis controlled for age, parity, multiple birth, insurance status; including random effects for place of delivery (hospital)

*** p-value<0.001; ** p-value<0.01; * p-value<0.05; (*) p-value<0.1
